# Molecular epidemiology of *Rhodococcus equi* in slaughtered swine, cattle and horses in Poland

**DOI:** 10.1186/s12866-016-0712-9

**Published:** 2016-05-27

**Authors:** Lucjan Witkowski, Magdalena Rzewuska, Shinji Takai, Magdalena Kizerwetter-Świda, Jerzy Kita

**Affiliations:** Laboratory of Veterinary Epidemiology and Economics, Faculty of Veterinary Medicine, Warsaw University of Life Sciences, Nowoursynowska 159c, 02-776 Warsaw, Poland; Department of Preclinical Sciences, Faculty of Veterinary Medicine, Warsaw University of Life Sciences, Ciszewskiego 8, 02-786 Warsaw, Poland; Department of Animal Hygiene, School of Veterinary Medicine, Kitasato University, Higashi 23-35-1, Towada, Aomori 034-8628 Japan

**Keywords:** *R. equi*, Swine, Equine, Meat, Slaughterhouse, Foodborne pathogen

## Abstract

**Background:**

*Rhodococcus equi* is an emerging zoonotic presumably foodborne pathogen. Since the data on the worldwide prevalence of *R. equi* in meat animals are scarce, the present study aimed to investigate the molecular epidemiology of *R. equi* in swine, cattle and horse carcasses intended for human consumption in Poland.

**Results:**

Totally 1028 lymph node samples were examined. *R. equi* was isolated from 26.6 % (105/395) swine and 1.3 % (3/234) bovine healthy submaxillary lymph nodes. In horses, *R. equi* was isolated only from 0.5 % (1/198) samples of middle tracheo-branchiales lymph node while no lymphocentrum retropharyngeum sample was positive (0/198). The purulent lesions were observed only in 0.8 % swine submaxillary lymph nodes samples (3/398) and in two of them *R. equi* was detected.

All bovine and most of swine isolates (98.1 %) were *vapB*-positive. 87.9 % of swine isolates carried 95-kb type 5 plasmid, 3.7 % type 1 and plasmid types: 4, 7, 10, 11, 21, 31 were carried by a single isolate (0.9 %). All bovine isolates carried VAPB type 26. Single horse isolate was *vapA*-positive and carried plasmid VAPA 85-kb type I.

**Conclusions:**

The prevalence of *vapB*-positive *R. equi* in investigated healthy swine intended for human consumption was very high.

Not only swine, but also even apparently healthy cattle or horse carcasses should be considered as a potential source of *R. equi* for humans, especially in countries where undercooked or raw beef or horsemeat is traditionally consumed.

## Background

*Rhodococcus equi* is a Gram-positive bacterium widespread in the environment of grazing farms. It is common in the feces of wildlife and farm animals including swine, cattle, horses and others [1–5]*.* The virulence of *R. equi* is determined by the virulence-associated plasmids (VAPs) which harbor the pathogenicity islands that contain *vap* genes encoding the virulence-associated proteins (Vaps) [6, 7]. In virulent strains isolated from horses the *vapA* gene encoding virulence-associated 15–17-kDa protein (VapA) is predominant, whereas in swine isolates the *vapB* gene of the virulence-associated 20-kDa protein (VapB) is most prevalent. Bovine isolates usually carry the *vapN* gene however some isolates carry the *vapB* gene. The aforementioned three genes are located on plasmids VAPA, VAPB, and VAPN, respectively. Interestingly, pVAPN is a 120-kb invertron-like linear replicon unrelated to the circular virulence plasmids associated with equine (pVAPA) and swine (pVAPB) and harbors new *vap* multigene family members (*vapN* to *vapS*). Avirulent, “plasmidless” strains, widespread in the environment, mainly in soil show no evidence of these plasmids but are positive for a *traA* plasmid conjugal transfer gene marker [8–10]. The “TRAVAP” typing scheme classifies *R. equi* isolates into 4 categories: *traA*^*+*^*/A*^*+*^*B*^*−*^ “horse-type”, *traA*^*+*^*/A*^*−*^*B*^*+*^ “pig-type”, *traA*^*+*^*/AB*^*−*^ “bovine-type” and *traA*^*−*^*/AB*^*−*^ plasmid-less type [10]. Using an unified nomenclature, plasmids were designated as pVAPA, pVAPB, and pVAPN (for “*noA*-*noB*”), respectively [7, 9]. Analysis of restriction enzyme digestion patterns revealed several distinct VAPA plasmid types and over 20 types of VAPB plasmid but the digestion patterns of VAPN have not been so far recognized [3, 11, 12].

In horses, *R. equi* is a well-known pathogen responsible for foal rhodococcosis – highly fatal disease typically manifesting itself as pyogranulomatous bronchopneumonia. The disease usually affects foals aged up to six months whereas it rarely occurs in adult horses [13, 14]. Sporadically, the bacterium can prove pathogenic for other animals. In swine, *R. equi* was isolated for the first time in 1930s of the XX century, from purulent lesions in lymph nodes [15]. Recent studies have revealed that *R. equi* not previously suspected *Mycobacterium* spp*.*, is the primary causal agent of lymphadenitis [16–21]. The disease was rarely described in ruminants such as camels [22], llamas [23] and mostly in goats [24]. Reports from cattle are sparse and all describe isolation from purulent lesions in the lymph nodes, mostly in animals with suspected tuberculosis [20, 25–27]. In wild animals *R. equi* was isolated from purulent lesions in the lymph nodes of wild boar [11, 28] and American bison as a co-infection with *Mycobacterium avium* subsp. *paratuberculosis* [29]. The clinical cases of pyogranulomatous skin disease and pneumonia associated with *R. equi* infection were also occasionally observed in cats and dogs [30].

Recently, *R. equi* infection in other species than horse has aroused considerable interest due to its frequent isolation from the lymph nodes and other tissues of apparently healthy animals intended for human consumption. The prevalence of *R. equi* in swine carcasses varies from 0 % to over 20 % [1, 2, 11, 18–20, 28, 31–33]. In wild boar the prevalence ranges from 0 % to even 52 % hunted animals [34–37], whereas in the roe deer and red deer it is lower than 1 % [37]. However, the data on the occurrence of *R. equi* in slaughtered apparently healthy cattle or horses are lacking.

The increasing number of *R. equi* infections in humans has been observed for several years and now *R. equi* is considered an emerging zoonotic pathogen. The clinical cases in humans are rare and so far have been usually diagnosed in immunocompromised patients [38]. Sources and routes of human infection remain unclear. It has been suggested that the contact with farm animals or their environment may plays a role in some cases of infection but a foodborne transmission seems to be the most likely route, especially the exposure by the consumption of raw, undercooked or contaminated meat [5, 10, 38, 39]. This theory is supported by the epidemiological relationship between human and animal *R. equi* infections. The strains of swine or bovine type have been isolated from human cases more often than equine or avirulent environmental strains [3, 8, 10, 39]. Furthermore, a pattern of geographical distribution of strains is similar in humans and animals. For example VAPB type 5 is predominant among isolates from humans and animals in Europe [12, 36], type 8 in the South America [5, 11, 39], while type 1 and 2 in Asia [1, 35].

Regardless of the significant progress, relatively little is known about types of virulence plasmids of *R. equi* isolated from the meat animals. The problem is important, as there is increasing trend for diagnosing *R. equi* infections in humans and growing rate of *R. equi* isolation from tissues of slaughtered or hunted animals. These facts may result from the infection running rife as well as from increasing awareness and improved diagnostics.

Thus, the aim of this study was to estimate the prevalence of *R. equi* in swine, cattle and horse carcasses intended for human consumption in Poland and characterize virulence plasmid types in the isolates.

## Results

The results of *R. equi* isolation are presented in Table 1. Totally, 107 *R. equi* strains were isolated from swine, 105 strains from lymph nodes without any purulent lesions (26.6 % of swine) and 2 other from lymph nodes with purulent lesions. At least several colonies of *R. equi* were observed in all positive cultures from swine samples. The purulent lesions (which varied in size from 1 to 5 millimeters in wide) were observed only in 3 (0.8 %; CI 95 %: 0.3–2.2 %) of samples and two of them were *R. equi* positive. No purulent lesions were observed in any of the samples from cattle or horses. *R. equi* in horses was isolated only once from the middle tracheo-bronchial lymph nodes of a two year-old stallion. The results of detection of plasmid genes *vapA* and *vapB* are presented in Table 1. The *choE* gene specific for *R. equi* was detected in all 111 isolates. All cattle and 98.1 % of swine isolates were *vapB*-positive. A single horse isolate was *vapA*-positive. Ten distinct VapB plasmid types were found in 10 *traA*-positive isolates: 9 in swine and 1 in cattle (Table 2). VAPB 95-kb type 5 was most prevalent in swine isolates and was detected in 94 of the *vapB*-positive isolates. All cattle isolates carried plasmid type 26. The horse strain carried 85-kb type I plasmid (Table 2).

**Table 1:** Prevalence of *R. equi* in lesion-free lymph nodes of slaughtered swine, cattle and horses

Animal species (specimen)	No. of samples	No. of isolates	Prevalence (%)	95 % CI (%)	No. of VapA positive isolates	No. of VapB positive isolates	% of Vap positive isolates
Swine (submaxillary lymph nodes)	395	105	26.6	22.5–31.1	0	103	98
Cattle (submaxillary lymph nodes)	234	3	1.3	0.5–3.7	0	3	100
Horse (lymphocentrum retropharyngeum)	198	0	0	0	0	0	0
Horse (middle tracheo-branchiales lymph nodes)	198	1	0.5	0.1–2.8	1	0	100
Total	1025	109			1	106

**Table 2:** Plasmid profiles of *R. equi* isolates from slaughtered swine, cattle and horses in Poland

animal species	virulence-associated plasmid	plasmid type	No. of isolates (total No.111)	% of isolates
Swine	VAPB	1	4	3.7
		4	1	0.9
		5	94	87.9
		6	1	0.9
		7	1	0.9
		10	1	0.9
		11	1	0.9
		21	1	0.9
		31	1	0.9
		plasmid-less	2	1.9
Cattle	VAPB	26	3	100
Horses	VAPA	85-1	1	100

## Discussion

Our results confirmed the presence of *R. equi* in submaxillary lymph nodes of apparently healthy swine intended for the human consumption. Moreover, the prevalence of *R. equi* in healthy swine (26.58 %) appeared very high, when compared with the data from most previous studies which reported 0 to 3 % [1, 16, 28, 31, 32]. Only two reports, one from Hungary [33] and one from Japan [2] presented the results closer to our findings (14 and 21 %, respectively).

It is interesting why *R. equi* prevalence among swine from different countries varied so much. The methodology of *R. equi* isolation from swine was similar in all the aforementioned studies. For example, CAZ-NB used in our study was widely used in similar studies and is commonly considered as the best selective medium for the isolation of *R. equi* [5]. It has been suggested that contamination of slaughtered swine carcasses with pathogenic *R. equi* might occur through feces [5]. It didn’t occur in this study due to food safety and quality procedures in the slaughterhouse and moreover, lymph nodes were collected with surrounding tissues and separated in the laboratory in sterile conditions. The *R. equi* infection in humans is usually diagnosed in immunocompromised patients [38]. In animals (wild boars and cattle) the prevalence is higher in young than in older ones [27, 35]. Perhaps in swine also some factors predisposing for *R. equi* infection exist, however they are not known.

Granulomatous lesions were rare in the investigated submaxillary lymph nodes from swine and *R. equi* was isolated in two of three samples. This finding corresponds with the previous studies reporting such lesions in approx. 0.5 % of lymph nodes [16, 21]. The first *R. equi* isolation from granulomatous lesions in lymph nodes in swine was described over 50 years ago, but its role still remains unclear [15]. One study revealed a very high prevalence of *R. equi* in submaxillary lymph nodes with purulent lesions, the bacteria alone was isolated from 45 % and together with *Mycobacterium avium* subsp. *avium* from next 55 % of samples [16]. Most of the studies reported isolation of *R. equi* alone from approx. 20 % of samples and together with mycobacteria from next 5 % of samples [19–21, 28]. The examination of lymphoid tissue from various organs of slaughtered swine with suspected *Mycobacterium* spp. infection showed similar proportion of *R. equi* infections alone (18 %), but much higher proportion of the mixed infections with mycobacteria (62 %) [17]. Moreover, the data on the role of *R. equi* in lymphadenitis in wild boar are inconclusive. Although in Brazil *R. equi* was isolated from 6.6 % of wild boar lymph nodes with lymphadenitis [28], no *R. equi* was isolated from purulent lesions in Poland [37]. Similarly, the studies dealing with the prevalence of *R. equi* in lesion-free wild boar submaxillary lymph nodes do not lead to clear conclusions. The reported prevalence varied from 0 % in Brazil [28], 5 % in Poland [37], 12 % in Hungary [34] to 52 % in Japan [35].

In our study almost all *R. equi* isolates obtained from swine were *vapB*-positive. Likewise, high prevalence of intermediate virulent strains in swine was described previously [31, 32]. However, other studies on swine isolates reported much lower percentage of *vapB*-positive strains, in approx. 30 % [18, 33] or 70 % of isolates [1, 19]. The *vapB*-positive *R. equi* strains accounted for 25 to 70 % of isolates in wild boar [11, 34–36]. Interestingly, only in Japan the *vapA*-positive *R. equi* strains typical for the horse were rarely isolated also from swine and wild boar [32, 35].

Our results revealed the presence of 95-kb type 5 plasmid in most of *vapB*-positive swine isolates. This plasmid type seems to be specific for *R. equi* isolates from Europe because it has thus far been detected in most of European swine and wild boar isolates [18, 34, 36]. The 95-kb type 1 VAPB plasmid, detected in 3.7 % of investigated swine strains in our study, was detected with similar frequency in wild boar *R. equi* isolates in Hungary [34] and swine isolates in Brazil [11]. In contrast, this VAPB type is predominant in Asia where was detected in approx. 30 % of swine and wild boar isolates [31, 35]. Other VAPB types of investigated *R. equi* swine isolates (0.9 % of investigated isolates each) were described in 5 to 10 % of cases, worldwide: type 4 in wild boar isolates in Japan [35], type 6 and 7 in swine isolates in Thailand [31], type 7 and 11 in wild boar isolates in Poland [36], type 10 in swine isolates in Brazil [11] and type 21 in wild boar and swine isolates in Hungary and Slovenia [18, 34]. Type 8 was not detected in any of investigated isolates. However, this type is predominant in swine and wild boar isolates in Brazil [5, 11].

*R. equi* seems to have minor clinical importance in cattle. Nevertheless, it was isolated (alone or together with mycobacteria) from 1–3 % [20, 26] or even 23.5 % [27] of lymph nodes with lesions in slaughtered cattle suspected of *Mycobacterium* spp. infection. Currently, *Mycobacterium* spp. infection is an important problem in some countries, but granulomatous lesion in bovine lymph nodes are observed rarely. Thus, the prevalence of *R. equi* in population of slaughtered cattle was described as very low [27]. However, the isolation of *R. equi* from 1.3 % of lesion-free lymph nodes in our study contradicts previous data. Furthermore, all 3 bovine isolates were *vapB*-positive and carried plasmid type 26, found previously in isolates from lesion-free lymph nodes of a wild boar in Hungary [34]. Unfortunately, it is difficult to compare the molecular data of *R. equi* epidemiology in cattle because virulence of isolates was investigated only in a few studies on meat animals and plasmid types were rarely defined [20, 26]. No *vapB*-positive strains were detected and from the cases of lymphadenitis in cattle in Ireland [27] and Germany [25]. Surprisingly 9 % of *R. equi* strains isolated from cattle feces in Germany were *vapA*-positive [4]. However, further analysis of 25 bovine strains from Ireland [27] showed that 72.0 % of isolates had the specific plasmid type associated with the bovine host *traA*^+^/*vapAB*^−^ (pVAPN in new nomenclature) [10].

All samples examined in our study were collected in slaughterhouses during a normal work day. Whole sampling procedure had to be non-disturbing and adapted to routine procedures in the facility. Submaxillary lymph nodes were easily available in swine and cattle carcasses but were not available in horses because were removed with the skin during flaying. Therefore, in horses we decided to collect lymphocentrum retropharyngeum as a lymph node representative for the head region. Additionally, we decided to collect middle tracheo-bronchial lymph nodes. Interestingly, in this study no *R. equi* strains were isolated from lymphocentrum retropharyngeum of horses and only a single *R. equi* strain was isolated from middle tracheo-branchiales lymph nodes. This is surprising, because horse is the only animal species in which *R. equi* infection poses a serious clinical problem. However, only foals up to six months of age have a unique susceptibility to clinical disease, and the clinical cases in adult horses were described very rare. Those cases are probably associated with the immunosuppression and activation of persistent infection of *R. equi* in lymph nodes [13, 14]. Thus, much higher prevalence of *R. equi* in investigated equine samples was expected. Isolated strain contains 85-kb type I VAPA plasmid which has been found in most of isolates from horses in Europe [12, 40].

## Conclusions

This study confirms the occurrence of *R. equi* in healthy swine intended for human consumption. However, the very high prevalence of *vapB*-positive *R. equi* strains in investigated apparently healthy animals rises a questions of predisposing factors for *R. equi* infection in swine.

To the best of authors’ knowledge this is the first report of *R. equi* isolation from slaughtered, apparently healthy cattle and the first report of *R. equi* in meat horses at all. The low prevalence of *R. equi* in lymph nodes in these species suggests that their meat is unlikely to be the main source of *R. equi* infection for humans

Not only swine, but also even apparently healthy cattle or horse carcasses should be considered as a potential source of *R. equi* for humans, especially in countries where undercooked or raw beef or horsemeat is traditionally consumed.

## Methods

The study was approved by the 3rd Local Commission for Ethics in Animal Experiments (Decision No. 44/2009). The population of investigated animals was estimated on the basis of the data of The Agency for Restructuring and Modernization of Agriculture, Central Statistical Office in Poland and Polish Horse Breeders Association. Sample size was designed for each animal species according to a commonly used epidemiological method [41]. In order to estimate a prevalence with an accepted error of 5 % in swine, and 10 % in cattle and horses (level of confidence of 95 %, expected prevalence of 50 %). A 95 % confidence interval (95 % CI) for proportions was calculated using Wilson score method. The calculations were performed in Win Episcope 2.0 [41]. In 2011 year 19 675 000 swine, 1 372 000 cattle and 60 000 horses were slaughtered in Poland.

Samples were collected in 3 slaughterhouses from carcasses accepted for human consumption in 2011 and 2012. Submaxillary lymph nodes were obtained from swine (*n* = 398) and cattle (*n* = 234). From horses (*n* = 198) lymphocentrum retropharyngeum and middle tracheo-bronchial lymph nodes were collected. A total number of 1028 lymph node samples were evaluated: 398 from swine, 234 from cattle and 396 from horses. The animals originated from whole territory of Poland. The swine were bred on industrial farms in closed buildings. The cattle and horses were of various breeds and in various age. Unfortunately, the detailed data was sometimes not available and therefore the place of origin and the age of animals could not be identified in several samples. The material was stored in −20 °C until further investigation.

*R. equi* isolation, phenotypic and genotypic identification of isolates was conducted as described previously [36]. Briefly, thawed lymph nodes were cut into small pieces using sterile scissors. Then, one gram of tissue was added to 3 ml of sterile 0.9 % saline and was homogenized using PRO200 homogenizer Multi-Gen 7 (PRO Scientific Inc., USA). Finally, 100 μl of homogenized tissue was cultured in selective medium. For the bacteria isolation modified CAZ-NB medium was used, biochemical properties of isolates were determined using API Coryne test (bioMerieux, France) and the presence of “equi factor” was studied in CAMP test. The presence of four *R. equi* genes, *choE, traA, vapA* and *vapB*, was determined by PCR.

Plasmid DNA was isolated from the *vapA* and *vapB*-positive *R. equi* isolates using an alkaline lysis method with some modification and digested with restriction endonucleases EcoRI, EcoT221, HindIII and BamHI as described previously [36].

## Abbreviations

VAP, virulence-associated plasmid; Vap, virulence-associated protein.
